# Randomized, open-label, crossover trial comparing the pharmacokinetic profile of a novel oral aspirin solution and a chewed aspirin tablet 

**DOI:** 10.5414/CP204271

**Published:** 2022-08-18

**Authors:** Dan Atar, Sougat Sarkar, Emil Kolev, Carla Mura, Frank Brosstad, Lotte Theodorsen, Geir Ivar Westen, Per Erik Stribolt-Halvorsen

**Affiliations:** 1 Institute of Clinical Medicine, University of Oslo, Institute for Clinical Sciences,; 2 Department of Cardiology, Oslo University Hospital Ullevål, Oslo, Norway,; 3 PLG group, Mumbai, India,; 4 Diagnostics and Consultation Center (DCC) Convex EOOD, Sofia, Bulgaria,; 5 Anapharm Bioanalytics, Barcelona, Spain,; 6 Department of Medicine, University of Oslo, Oslo, and; 7 Asamedic AS, Trollåsen, Norway

**Keywords:** acetylsalicylic acid, ASA, salicylic acid, formulations, fasting, fed

## Abstract

Objectives: The primary objective of this study was to assess the pharmacokinetic profiles of acetylsalicylic acid (ASA) and salicylic acid (SA) after administration of two different formulations of aspirin under fasting and fed conditions. Materials and methods: The study was a randomized, open-label, parallel-group, 2-arm crossover study conducted at a single center. Healthy subjects were randomized to receive 300 mg of aspirin in either a 15-mL oral solution (pre-packaged vial containing powder and solvent that are combined at the time of administration) or a single solid tablet to be chewed and swallowed with 150 mL of water. Treatment visits were separated by a 10-day wash-out period. Results: At 3 minutes, ASA concentrations for the oral solution fed state and fasting state arms exceeded those for the chewed tablet (fed 299 vs. 139 ng/mL; fasting 356 vs. 204 ng/mL). Compared to the chewed tablet, the mean plasma ASA concentration was 74% greater with the oral solution under fasting conditions, and 115% greater under fed conditions. Similarly, at 3 minutes, the mean SA plasma concentration with the oral solution under fed and fasting conditions exceeded those for the chewed tablet (fed 310 vs. 160 ng/mL; fasting 330 vs. 185 ng/mL). Under fasting conditions, the mean plasma ASA AUC_0-last_, with the oral solutions was 168,076.8 min.ng/mL compared to 163,726.3 min.ng/mL with the chewed tablet. Under fed conditions, the mean plasma ASA AUC_0-last_, with the oral solutions was 179,116.7 min.ng/mL compared to 164,704.3 min.ng/mL with the chewed tablet. Conclusion: This phase 1 study showed that use of an aspirin oral solution provided more rapid exposure to higher plasma concentration levels of ASA and SA than chewing a solid tablet.

**What is known about this subject**

- Quick administration of aspirin is vital for improving survival during suspected myocardial infarction
- The aspirin formulation and ingestion method can impact the time to inhibition of platelet aggregation
- Studies have shown that chewing aspirin tablets has a faster onset compared to swallowing whole tablets

**What this study adds**

- A novel oral aspirin solution provided more rapid exposure to higher plasma concentration levels of ASA than chewing a solid tablet
- ASA concentrations for the oral solution exceeded those for the chewed tablet in both the fed state and fasting states
- Clinically, early administration of aspirin has been associated with better outcomes than later administration, and this study suggests that a potential benefit may be associated with an oral aspirin solution

## Introduction 

Aspirin is an integral component of the acute management of suspected myocardial infarction (MI) [1, 2]. In patients with acute MI undergoing thrombolysis, early use of aspirin after symptom onset (median 1.6 hours) was associated with a significant reduction in mortality at 7 days, 30 days, and 1 year compared to later administration (median 3.5 hours), with adjusted odds ratios of 0.36, 0.39, and 0.41, respectively [3]. Therefore, in the acute situation, it is important to rapidly achieve platelet inhibition. The time to achieve sufficient plasma levels of acetylsalicylic acid (ASA) to inhibit platelet aggregation is impacted by the aspirin formulation and the ingestion method [4, 5, 6, 7, 8, 9, 10]. 

Most studies have compared the effects of chewing aspirin tablets to those that are swallowed whole. As a result, current clinical guidelines recommend patients receive a rapid loading dose of 150 – 300 mg of aspirin, and that tablets should be chewed rather than swallowed intact [1]. However, alternative formulations may be able to further increase the speed of ASA-induced platelet inhibition beyond what is seen with chewing. Soluble aspirin formulations (i.e., those swallowed as part of a liquid) have demonstrated significantly shorter median times to platelet inhibition compared to whole aspirin tablets [7], and some clinical guidelines recommend the use of dissolvable aspirin formulations [11]. 

The goal of this study was to assess the pharmacokinetic properties of an oral solution containing 300 mg of ASA to determine its feasibility for use as an emergency treatment for suspected acute MI. The study protocol was registered with the European Clinical Trials Database (EudraCT: 2020-03873-23). 

## Materials and methods 

### Study objectives 

The primary objective of this study was to assess the pharmacokinetic profiles of ASA and salicylic acid (SA) after administration of two different formulations of aspirin under fasting and fed conditions. Secondary objectives included various pharmacodynamic and safety assessments, which will be discussed in a separate report. 

### Study medications 

Study participants received 300 mg of ASA in either a 15-mL oral solution (pre-packaged vial containing powder and solvent that are combined at the time of administration) (Coxor, Asamedic AS, Kolbotn, Norway) or as a single solid, non-enteric-coated tablet (Polopiryna S, Polpharma OTC, Starogard Gdański, Poland) to be chewed and swallowed with 150 mL of water under both fasting and fed states. 

### Study subjects 

Subjects were healthy individuals, defined as those free from clinically significant illness or disease as determined by their medical history and physical examination, with no contraindications for the study medications. Individuals were 18 – 55 years, had a body mass index (BMI) of 18.5 – 30.0 kg/m^2^, and gave informed written consent. 

Exclusion criteria were: COVID-19 RT-PCR positive test at screening or at any treatment visit; general health condition requiring pharmacological treatment; known hypersensitivity to ASA, other salicylates, or non-steroidal anti-inflammatory drugs (NSAIDs); history of bleeding disorders, peptic ulceration, severe renal or hepatic impairment, infectious diseases, asthma or alcohol or drug use disorder; clinically significant abnormal laboratory values, elevated blood pressure; abnormal heart rate; fever; pregnant or breast-feeding at screening; or any other reason that the investigator deemed inappropriate for study participation. 

Subjects had to abstain from consumption of caffeine or other methylxanthines within 48 hours; grapefruit or other citrus juices within 72 hours; use of any over-the-counter medication within 7 days; use of any prescription drugs (especially NSAIDs, anticoagulants, and selective serotonin reuptake inhibitors) within 14 days; and alcohol consumption within 48 hours prior to product administration. 

### Study design 

The study was a single-center, randomized, open-label study, comprising 2 parallel groups, each completing a 2-arm crossover study. Healthy subjects were randomized in a 1 : 1 ratio to group 1 (fed state), or group 2 (fasting state), with the sequence of treatments (arm 1: chewed tablet, arm 2: oral solution) in each group also randomized. The study period comprised 61 days, from screening (–14 to –3 days prior to treatment visit 1), to treatment and study visits (days 0 – 1 and days 11 – 12), to final follow-up at 4 (± 1) weeks following treatment visit 2. Both treatment visits involved an overnight stay at the clinical facility and were separated by a 10-day wash-out period. 

Patients in the fed group received a high-fat breakfast (825 kcal) 30 minutes before administration of the study drug, and received lunch (1,170 kcal), snack (480 kcal), and supper (632 kcal) on the study day. The fasting group received a light meal immediately prior to fasting for 8 hours prior to administration of the study drug, and received a standardized meal 240 minutes after administration of the study drug. Water was allowed throughout the study with the exception of 1 hour prior and 1 hour after dosing, during which time only the water required for dosing was allowed. Decaffeinated tea and coffee were allowed from 4 hours post-dose. 

### Pharmacokinetic analysis 

Blood samples (4 mL/sample) for analysis of ASA and SA concentrations were drawn at 60 and 30 minutes prior to dosing, and at 1, 3, 5, 7, 10, 15, 20, 25, 30, and 45 minutes, and 1, 1.5, 2, 3, 4, 6, 8, 12 hours after dosing. Samples were collected via an indwelling catheter, which was flushed with isotonic saline after sample collection. The sera were separated by centrifugation, and were then frozen and stored at −80 °C until required for analysis. 

Serum ASA and SA concentrations were measured by Anapharm Bioanalytics (Barcelona, Spain) using a previously validated liquid chromatography tandem mass spectrometry method, in compliance with European Medicines Agency and Food and Drug Administration guidance for bioanalytical method validation. The column type was reversed phase, and the matrix was human EDTA K_2_ plasma. Analyses were conducted with Sciex (Framingham, MA, USA) software (analyst version 1.6.2). The method was validated prior to start of the analysis, using a calibration curve plus 6 replicates of low-, medium-, and high-quality control samples. The lower limit of quantitation was 10 ng/mL for ASA and 100.56 ng/mL for SA. All concentrations below the limits of quantitation of the assay are set to one-half of the limit of quantification. 

Pharmacokinetic parameters assessed included: maximum measured plasma concentration (C_max_), area under the plasma concentration curve from administration to last observed concentration at time t (AUC_0-last_), time until maximum plasma concentration is reached (t_max_), plasma elimination half-life (T_1/2_), elimination rate constant (K_el_), and residual area (%). 

### Statistical analysis 

Sample size was calculated based on expected values of pharmacokinetic parameters. Assuming a power of 80% and coefficient of variation of 10%, 11 participants would be needed to complete the study with point estimate of 90%. A final sample size of 24, divided between two parallel groups was calculated assuming a 10% drop-out rate. 

Statistical analysis and data tabulation was performed on a per-treatment basis, which included all subjects who received 1 dose of the test drug, did not have a major protocol deviation, and had sufficient plasma concentration-time profiles. Missing test results or assessments were not imputed, descriptive statistics and statistical analyses were performed on the basis of the available data only. Subjects who discontinued were included in the descriptive statistics if they received study treatments at the scheduled time. 

Pharmacokinetic parameters (C_max_, AUC_0-t_, AUC_0-∞_, t_max_, T_1/2_, K_el_) were calculated for each treatment using Phoenix WinNonlin v8.3 (Princeton, NJ, USA) and statistical analyses were conducted with SAS v9.4 (Cary, NC, USA). The pharmacokinetic endpoints were analyzed using non-compartmental methodology, and the results presented as mean values (± standard deviation) or as mean ratios with 90% confidence intervals (CI). All statistical tests (parametric and non-parametric) were interpreted at the 5% two-sided significance level. 

Analyses of variance (ANOVA) with fixed effects were performed on the natural log-transformed C_max_ and AUC_0-t_ using SAS/GLM procedures. Each ANOVA model included calculation of least-squares means (LSM), the differences between formulation LSM, and the standard error associated with these differences. Ratios of LSM were calculated and expressed as a percentage relative to the reference medication. 

## Results 

### Participants 

Healthy subjects were randomized to group 1 (fed state) (n = 12) or group 2 (fasting state) (n = 12). Three subjects in the fasting group discontinued after the first period and were not included in the pharmacokinetic analyses; 1 did not receive the oral solution (positive COVID-19 PCR test), and 2 did not receive the chewed tablet (1 withdrawal, and 1 positive COVID-19 PCR test). 

In the fed group, the mean age was 31.3 years (range 18 – 51), mean weight was 68.0 kg (range 47 – 94), and 50% were female. In the fasting group, the mean age was 29.2 years (range 22 – 36), mean weight was 73.6 kg (range 57 – 108), and 42% were female. Additional demographic data are presented in Table 1. 

### ASA concentration levels 

**Figure 1.**
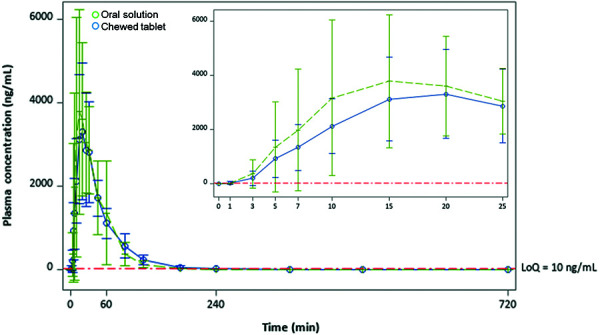
Mean (± SD) serum concentration-time profiles for acetylsalicylic acid after ingestion of aspirin as oral solution and chewed tablets during fasting state. LoQ = limit of quantification; SD = standard deviation.

**Figure 2.**
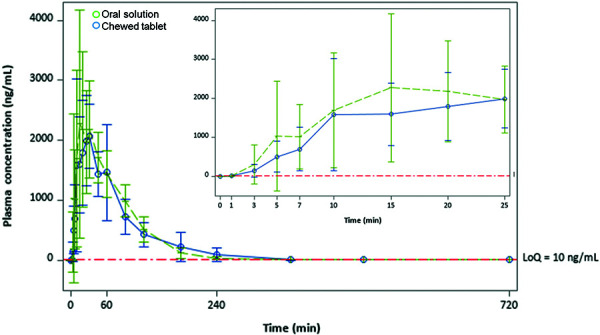
Mean (± SD) serum concentration-time profiles for acetylsalicylic acid after ingestion of aspirin as oral solution and chewed tablets during fed state. LoQ = limit of quantification; SD = standard deviation.

Plasma concentrations of ASA at multiple time points for both the oral solution and the chewed tablet, and for fed and fasting states, are shown in Figures 1 and 2. At 3 minutes, ASA concentrations were distinct and measurable, with the arithmetic means for the oral solution fed state and fasting state arms exceeding those for the chewed tablet (fed 299 vs. 139 ng/mL; fasting 356 vs. 204 ng/mL). Compared to the chewed tablet, the mean plasma ASA concentration was 74% greater with the oral solution under fasting conditions, and 115% greater under fed conditions. At 5 minutes, the mean ASA plasma concentration of the oral solution was 48% greater than the chewed tablet in the fasting group (1,347 vs. 913 ng/mL) and 104% greater in the fed group (1,027 vs. 504 ng/mL). At 10 minutes, the absolute difference between the oral solution and the chewed tablet peaked at 1,043 ng/mL (a 49% difference) in the fasting state. 

### SA concentration levels 

**Figure 3.**
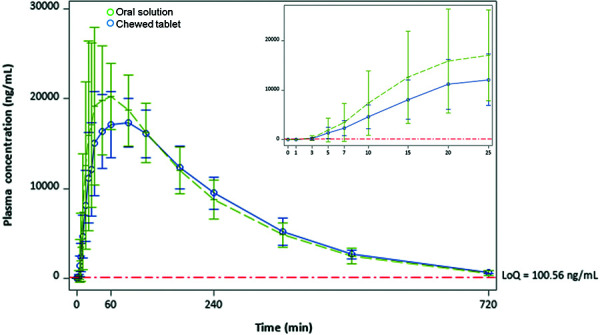
Mean (± SD) serum concentration-time profiles for salicylic acid after ingestion of aspirin as oral solution and chewed tablets during fasting state. LoQ = limit of quantification; SD = standard deviation.

Plasma concentrations of SA at multiple time points for both the oral solution and the chewed tablet, and for fed and fasting states, are shown in Figures 3 und 4. At 3 minutes, the arithmetic mean SA plasma concentration with the oral solution under fed and fasting conditions exceeded those for the chewed tablet (fed 310 vs. 160 ng/mL; fasting 330 vs. 185 ng/mL). This equates to increases in mean plasma SA concentrations of 94% and 78% with the oral solution compared to the chewed tablet for the fed and fasting conditions, respectively. At 5 minutes, the mean SA concentration with the oral solution was 38% greater than with the chewed tablet in the fasting state (1,904 vs. 1,383 ng/mL) and 100% in the fed state (1,380 vs. 689 ng/mL). At 10 minutes, the absolute difference in SA concentrations between the oral solution and the chewed tablet was 2,771 ng/mL (a 60% difference) in the fasting state. 

### Other pharmacokinetic parameters 

Mean t_max_ for ASA were similar in both the fed and fasting groups for both treatments at ~ 24 – 30 minutes (± 9 – 16 minutes). However, C_max_ was greater with oral solution compared to chewed tablet during both states, but especially during the fasting state (5,356 ng/mL vs. 3,592 ng/mL). Additional pharmacokinetic values are shown in Tables 2 and 3. 

## Discussion 

The present study showed that higher ASA concentrations can be obtained more rapidly with an oral solution compared with a solid tablet, despite being chewed, and provide support for use of an oral aspirin solution during suspected acute MI. The formulations were safe and well tolerated, with no unexpected safety issues. 

A number of studies have shown that chewing aspirin tablets resulted in more rapid ASA and SA levels than when the tablets are swallowed whole [4, 5, 6, 7, 8]. Few studies have compared oral solutions and chewed tablets. However, most studies, including this one, have reported higher ASA or SA concentrations were achieved more rapidly with soluble versus chewed aspirin formulations [7, 8, 9, 10]. These findings were confirmed in this study, which found that higher concentrations of both ASA and SA were achieved more rapidly with the oral solution, especially during the early time period. 

In the Feldman and Cryer study [4], an oral solution was included in the form of Alka Seltzer (Bayer Corp., Whippany, NJ, USA). The study found that more participants achieved detectable plasma ASA concentrations (85 ng/mL) within 3 minutes with chewed aspirin compared to the soluble form. In addition, the time to reach a 50% decrease in thromboxane B_2_ was longer with the soluble form (7.6 minutes) than with the chewed aspirin tablet (5 minutes) [4]. Schwertner et al. [7] also compared Alka Seltzer to intact and chewed aspirin tablets. They measured plasma SA levels, and in contrast to the Feldman and Cryer study, soluble aspirin was associated with more rapid, and higher concentrations than the chewed or whole aspirin. Both soluble and chewed aspirin inhibited platelet aggregation at a median of 7.5 minutes, with no difference between the two forms. This study also found superior results for ASA and SA levels with the oral solution over the chewed tablet, which may be related in part to differences in oral formulations. 

These studies also reported ASA or SA plasma concentrations that were associated with inhibition of platelet aggregation. Feldman and Cryer [4] reported a 50% decrease in thromboxane B_2_ at a plasma ASA concentration of 1,000 ng/mL, and Schwertner et al. [7] reported a plasma SA concentration of 2,460 ng/mL was associated with inhibition of platelet aggregation. While ASA concentrations are more important for antiplatelet effects, in clinical studies SA levels are often measured because of the rapid breakdown of ASA to SA. However, SA levels have been shown to correlate with platelet function assays making it a useful surrogate measure [12]. In this study, ASA concentrations over 1,000 ng/mL were achieved within 5 minutes with the soluble formulation in both the fasting and fed states, however, this level was not achieved with the chewed tablet until 7 minutes in the fasting state, and was further delayed (10 minutes) in the fed state. Similar to the Schwertner study, SA concentration of 2,460 ng/mL were achieved within 5 – 7 minutes with the oral solution in the fasting state in this study. 

These prior studies assessed pharmacokinetics in a fasting state. Studies show that aspirin absorption rate is significantly affected by food for various reasons [6, 13, 14], which was also seen in this study. Data in the fasting state may not always reflect the clinical situation, patients may develop acute symptom onset after a meal. Similarly, in this study, although lower levels of ASA and SA were seen in the fed compared to the fasting state with both formulations, the oral solution was associated with more rapid and higher peak concentrations compared to the chewed tablet in both conditions. This study was designed to compare the liquid and the tablet formulations, and not to directly assess the effects of food on aspirin pharmacokinetics. 

One limitation of the present study may be the lack of a treatment arm assessing aspirin swallowed whole; however, this was deemed unnecessary due to the preponderance of evidence demonstrating the superiority of chewed versus whole aspirin tablets. In addition, pharmacodynamic analyses were not complete at the time of this writing; however, these data will be presented in a future report. 

Clinical studies show that early administration of aspirin is associated with better outcomes than later administration, and guidelines recommend that patients receive a rapid loading dose of aspirin. Based on the desire for rapid inhibition of platelet aggregation to prevent ischemic injury, it is likely that minutes could matter. 

The results of this phase 1 study suggest that use of an aspirin oral solution can provide a more rapid exposure to higher plasma concentration levels of ASA and SA than other administration methods. This suggests that a potential benefit may be associated with an oral aspirin solution, but phase 3 clinical outcome studies are needed. 

## Acknowledgment 

The authors would like to thank Sameer Shelar (PLG group, Mumbai, India), Lilyana Mircheva (Diagnostics and Consultation Center Convex, Bulgaria), and Cintia Jimenez (Anapharm Bioanalytics, Spain) for help with data collection and analysis. We would also like to thank Pauline Lavigne and Steven Portelance (unaffiliated) for contributions to writing and editing the manuscript. 

## Clinical trial registration 

The study protocol was registered with the European Clinical Trials Database (EudraCT: 2020-03873-23). 

## Authors’ contributions 

All authors have made substantial contributions to the submitted work, either conducting the study, data analysis, or manuscript preparation/revision, and all have seen and approved the manuscript currently being submitted to the International Journal of Clinical Pharmacology and Therapeutics. 

## Funding 

This study was funded by Asamedic AS, Trollåsen, Norway. 

## Conflict of interest 

DA has been an advisor to Asamedic and has received stock-options. SS is an employee of PLG group. EK is an employee of the Diagnostics and Consultation Center Convex. CM is an employee of Anapharm Bioanalytics. FB has been an advisor to Asamedic. LT is a consultant hired by Asamedic. GIW is a full-time employee (manager) and shareholder of Asamedic. PESH was co-founder, and is an advisor and shareholder of Asamedic. 

**Table 1.** Baseline demographics of study participants.

Characteristic	Group 1 – Fed (n = 12)	Group 2 – Fasting (n = 12)
Mean age, y (SD)	31.3 (9.53)	29.2 (5.27)
Female sex, n (%)	6 (50)	5 (41.7)
Mean height, cm (SD)	171.3 (9.93)	175.3 (8.90)
Mean weight, cm (SD)	68.0 (14.23)	73.6 (13.54)
Mean BMI, kg/cm^2^ (SD)	23.0 (3.47)	23.8 (2.46)

BMI = body mass index; SD = standard deviation.

**Figure 4.**
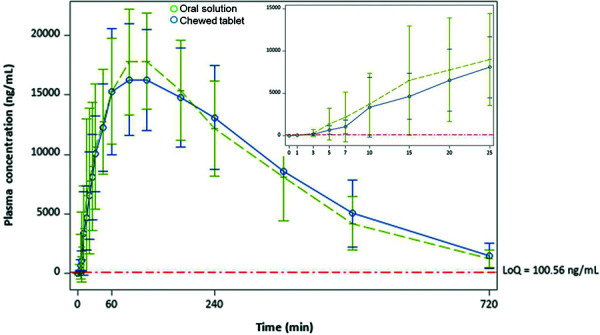
Mean (± SD) serum concentration-time profiles for salicylic acid after ingestion of aspirin as oral solution and chewed tablets during fed state. LoQ = limit of quantification; SD = standard deviation.

**Table 2.** Pharmacokinetic parameters of acetylsalicylic acid after ingestion of aspirin as oral solution and chewed tablets during fed and fasting states.

Parameter, mean (SD)	Group 1 – Fed		Group 2 – Fasting	
	Oral solution (n = 12)	Chewed tablet (n = 12)	Oral solution (n = 11)	Chewed tablet (n = 10)
Mean C_max_, ng/mL	2,797.48 (1,759.13)	2,539.03 (1,190.22)	5,356.36 (1,913.31)	3,592.24 (1,464.05)
Mean t_max_, min	24.17 (10.41)	29.58 (13.05)	25.91 (15.62)	23.50 (9.44)
Mean AUC_0-last_, min×ng/mL	179,116.7 (42,527.27)	164,704.3 (59,165.95)	168,076.8 (32,236.91)	163,726.3 (44,903.45)
Mean AUC_0-∞_, min×ng/mL	180,681.7 (43,011.80)	178,642.5 (54,004.47)	168,806.8 (32,232.45)	164,557.4 (44,776.97)
Mean T_1/2_, min	28.86 (5.79)	34.32 (11.97)	22.01 (4.96)	24.11 (4.50)
Mean K_el_, 1/min	0.0249 (0.0048)	0.0222 (0.0066)	0.0329 (0.0072)	0.0296 (0.0051)

C_max_ = maximum measured plasma concentration; AUC_0-last_ = area under the plasma concentration curve from administration to last observed concentration at time t; t_max_ = time until maximum plasma concentration is reached; T_1/2_ = plasma elimination half-life; K_el_ = elimination rate constant.

**Table 3.** Pharmacokinetic parameters of salicylic acid after ingestion of aspirin as oral solution and chewed tablets during fed and fasting states.

Parameter, mean (SD)	Group 1 – Fed		Group 2 – Fasting	
	Oral solution (n = 12)	Chewed tablet (n = 12)	Oral solution (n = 11)	Chewed tablet (n = 10)
Mean C_max_, ng/mL	18,657.96 (4711.94)	17,541.99 (4,903.33)	24,260.92 (3,997.36)	18,512.14 (3,395.98)
Mean t_max_, min	99.17 (29.06)	115.00 (50.90)	43.18 (27.95)	66.00 (22.58)
Mean AUC_0-last_, min×ng/mL	5,901,049 (1,839,576)	6,017,540 (2,064,588)	5,001,960 (901,000)	4,875,726 (822,067.4)
Mean AUC_0-∞_, min×ng/mL	6,155,759 (1,987,591)	6,334,133 (2,276,641)	5,106,197 (925,389.5)	4,997,726 (820,878.4)
Mean t_1/2_, min	133.38 (17.64)	138.55 (21.10)	119.44 (18.54)	122.75 (17.83)
Mean K_el_, 1/min	0.0053 (0.0007)	0.0051 (0.0008)	0.0059 (0.0009)	0.0058 (0.0009)

C_max_ = maximum measured plasma concentration; AUC_0-last_ = area under the plasma concentration curve from administration to last observed concentration at time t; t_max_ = time until maximum plasma concentration is reached; T_1/2_ = plasma elimination half-life; K_el_ = elimination rate constant.
